# Supplementary material for: The Study of Enteromorpha-Based Reinforcing-Type Flame Retardant on Flame Retardancy and Smoke Suppression of EPDM
Source: Polymers (Basel). 2022 Dec 23;15(1):55. doi: 10.3390/polym15010055 (PMC9823468; doi:10.3390/polym15010055)
Supplement: Supplementary file 1 [file polymers-15-00055-s001.zip › polymers-2073817-supplementary.pdf]

The Study of Enteromorpha Based Reinforcing Type Flame Retardant on Flame Retardant and Smoke Suppression of EPDM

Peipei Sun [1], Ziwen Zhou [2], Licong Jiang [2], Shuai Zhao [2,*], Lin Li [2,*]

1 Advanced Materials Institute, Qilu University of Technology (Shandong Academy of Sciences, Jinan 250014, China

2 Key Lab of Rubber-plastics, Ministry of Education/Shandong Provincial Key Lab of Rubber-plastics, School of Polymer Science and Engineering, Qingdao University of Science and Technology, Qingdao 266042, China.

＊ Correspondence: lyzhsh@163.com (S.Z.); qustlilin@hotmail.com (L.L.)

Table S1 The batch compositions for AEG system.

Fire retardant	APP(g)	EN	GE	TA
AEG20	20	20	1	0.1
AEG30	30	20	1	0.1
AG40	40	--	1	0.1
AE40	40	20	--	--
AEG40	40	20	1	0.1

Table S2 Table 2 LOI tests experimental data for various samples.

Samples	EPDM (phr)	TGE (phr)		EN (phr)	LOI (%)
		GE (phr)	TA (phr)		
Neat EPDM	100	--	--	--	24.1
EPDM/EN5	100	--	--	5	24.5
EPDM/EN10	100	--	--	10	25.1
EPDM/EN20	100	--	--	20	26.1
EPDM/EN30	100	--	--	30	25.8
EPDM/TGE0.5	100	0.5	0.05	--	24.7
EPDM/TGE1	100	1	0.01	--	25.5
EPDM/AEG	100	1	0.01	20	26.8

Table S3 Cone calorimetry experimental data for EPDM/APP40.

Samples	TTI (s)	PHRR (kW/m^2)	THR (MJ/m^2)	TSP (m^2)	MASS (g)	FIGRA ($kW/(m^2/s)$)	FPI ($10^{-2}m^2s/kW$)
EPDM/APP40	62	380.2	120.5	10.0	18.6	1.87	16.3

Table S4 Mechanicl properties for EPDM/APP40.

Sample	Item				
	Tensile strength /MPa	Elongation at break %	100% Modulus /MPa	300% Modulus /MPa	Tear strength N/mm
EPDM/APP40	6.64	282.45	2.15	6.77	38.16

Table S5 Raman results of EPDM/AEG composites.

Samples	Peak D/cm^{-1}	Peak G/cm^{-1}	I_D/I_G
Neat EPDM	240.92	299.92	3.76
EPDM/AEG20	237.11	274.72	3.51
EPDM/AEG30	365.36	408.65	3.25
EPDM/AG40	208.72	236.82	3.24
EPDM/AE40	247.94	278.51	2.99
EPDM/AEG40	249.40	266.16	2.59
